# Tooth Ache

**Published:** 1847-10

**Authors:** R. C. Carter


### *To Cure the Tooth-Ache.*

The patient is enjoined not to narrate what is done to him, or the pain will return, (but a repetition will restore the cure;) bah!

All the finger and toe nails are to be trimmed, the pieces off of each are to be lain on a rag or paper ; to which also is to be lain a lock of hair taken from the head; then the gum of the tooth is to be gouged or pierced, to add some blood to the nails and hair ; then the whole is to be wrapped together in the bank of some creek or gulley, at a place where no creature crosses. The operator may keep the putting away to himself if he pleases.

Respectfully, yours,

To Professor Taylor. R. C. CARTER.

The above, handed in by Dr. Carter, is one of the peculiar ways which ignorance and superstition plays upon the credulity of suffering humanity; to show what ignorance will lead to, we may give, in future, many such methods of cure—those having faith can use them if they desire.—Cin. Ed.

